# Supplementary material for: Identification of tolerance levels on the cold-water coral Desmophyllum pertusum (Lophelia pertusa) from realistic exposure conditions to suspended bentonite, barite and drill cutting particles
Source: PLoS One. 2022 Feb 22;17(2):e0263061. doi: 10.1371/journal.pone.0263061 (PMC8863230; doi:10.1371/journal.pone.0263061)
Supplement: S1 Appendix — (DOCX) [file pone.0263061.s001.docx]

# Revision - PONE-D-21-17662

“Identification of tolerance levels on the cold-water coral Desmophyllum pertusum (Lophelia pertusa) from realistic exposure conditions to suspended bentonite, barite and drill cutting particles” by Baussant et al.

# Supplemental material

**S1 Appendix.**


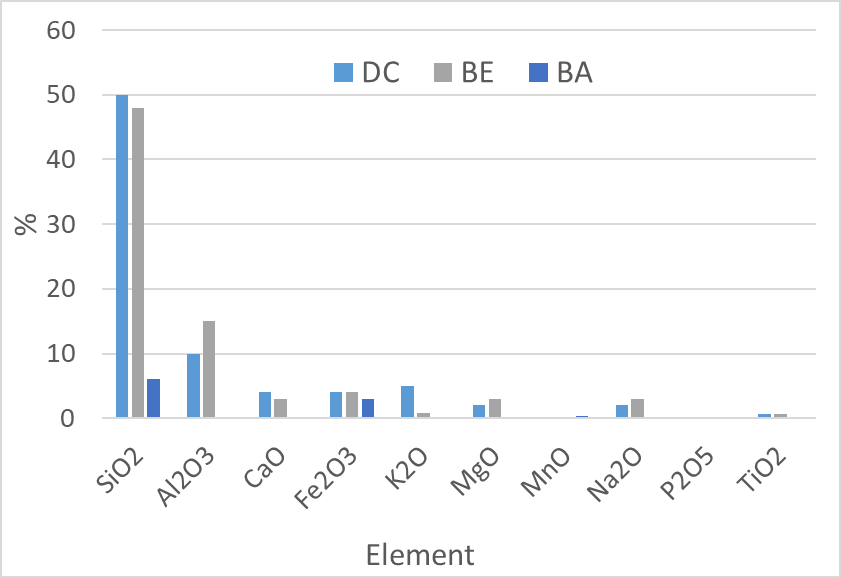
A)


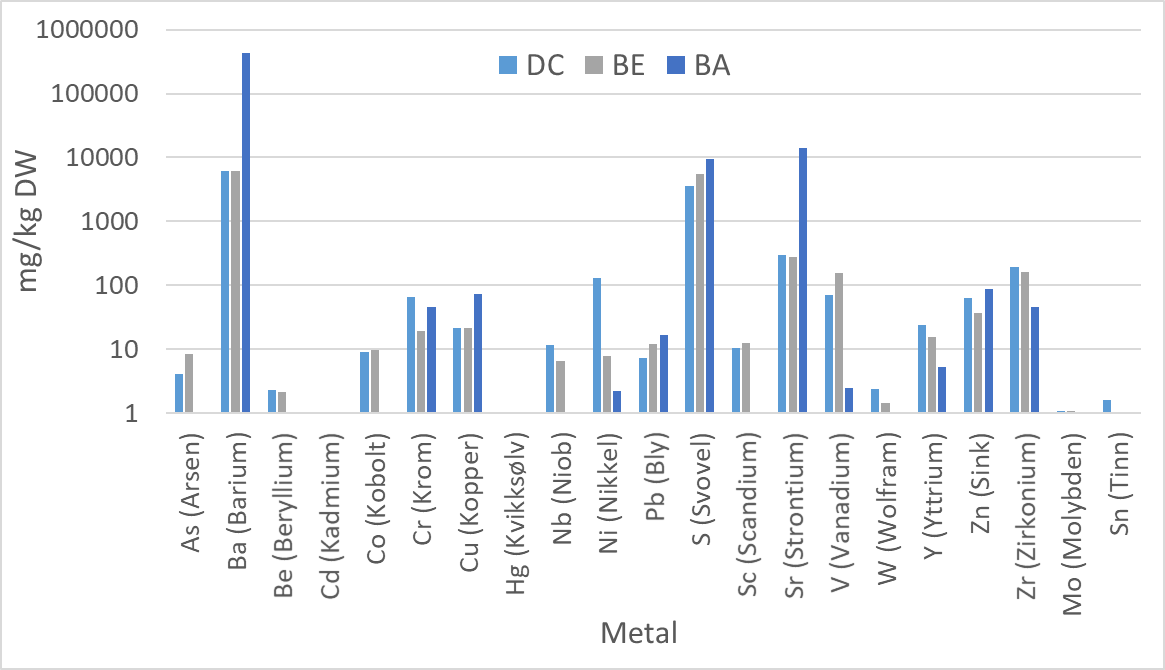
B)


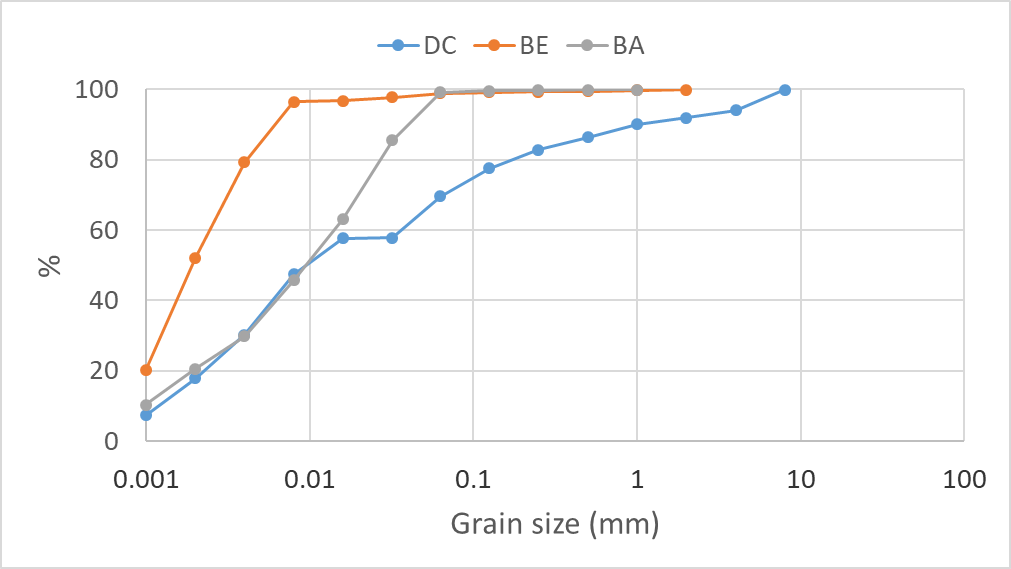
C)
