# Supplementary material for: Identification of tolerance levels on the cold-water coral Desmophyllum pertusum (Lophelia pertusa) from realistic exposure conditions to suspended bentonite, barite and drill cutting particles
Source: PLoS One. 2022 Feb 22;17(2):e0263061. doi: 10.1371/journal.pone.0263061 (PMC8863230; doi:10.1371/journal.pone.0263061)
Supplement: S4 Appendix — DT1T0 is the skeleton growth rate measured from T0 (prior to exposure) to T1 (2 weeks recovery), and DT2T1 is the skeleton growth rate measured between T1 an T2 (6 weeks recovery). Nominal concentrations are used on X-axis. The line in the box is the median value, and whiskers are the highest and lowest data below 1.5xIQR. Outliers are visualized with circles and extreme values with stars. (DOCX) [file pone.0263061.s004.docx]

# Revision - PONE-D-21-17662

“Identification of tolerance levels on the cold-water coral Desmophyllum pertusum (Lophelia pertusa) from realistic exposure conditions to suspended bentonite, barite and drill cutting particles” by Baussant et al.

# Supplemental material

**S4 Appendix.**


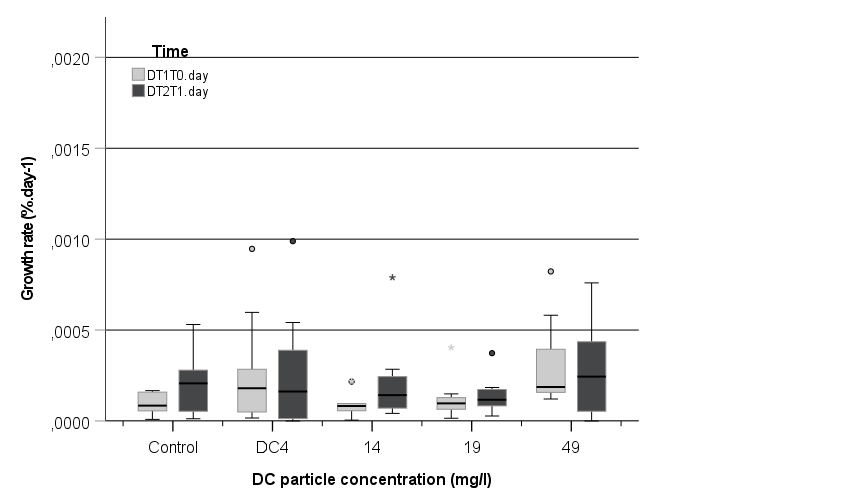
A)


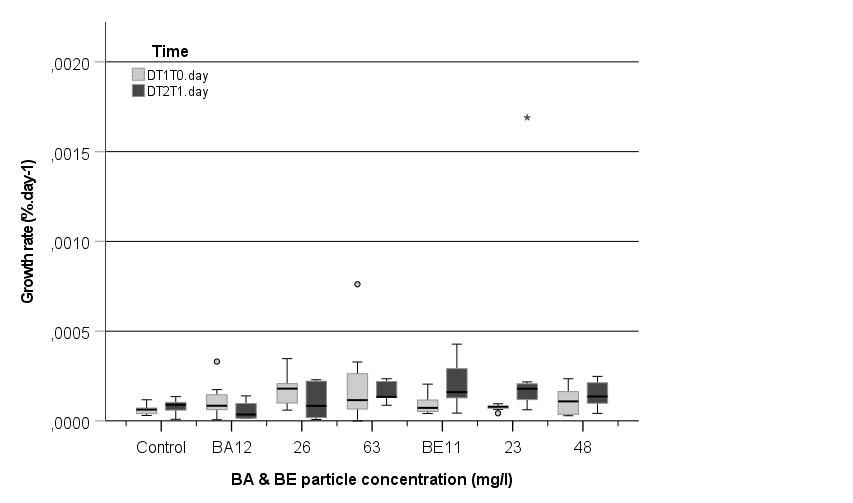
B)
