# Supplementary material for: Identification of tolerance levels on the cold-water coral Desmophyllum pertusum (Lophelia pertusa) from realistic exposure conditions to suspended bentonite, barite and drill cutting particles
Source: PLoS One. 2022 Feb 22;17(2):e0263061. doi: 10.1371/journal.pone.0263061 (PMC8863230; doi:10.1371/journal.pone.0263061)
Supplement: Attachment — Submitted filename: Authors Response.docx [file pone.0263061.s005.docx]

PONE-D-21-17662

[em.pone.0.76f0cb.b2d7b204@editorialmanager.com](mailto:em.pone.0.76f0cb.b2d7b204@editorialmanager.com)

Dear editor of PlosOne,

We thank you for the reviewing process and attach herein the responses to each point raised by the academic editor and the reviewer.

We have prepared a manuscript version with TRACK changes and one CLEAN version to visualize where in the text changes were made.

We hope these responses are complete and will help to make a final decision for publication of our manuscript in PlosOne.

Best regards

Thierry Baussant

==================================================================================

**Journal Requirements:**

1. Please ensure that your manuscript meets PLOS ONE's style requirements, including those for file naming. The PLOS ONE style templates can be found at 
<https://journals.plos.org/plosone/s/file?id=wjVg/PLOSOne_formatting_sample_main_body.pdf> and 
<https://journals.plos.org/plosone/s/file?id=ba62/PLOSOne_formatting_sample_title_authors_affiliations.pdf>

2. Please review your reference list to ensure that it is complete and correct.

This has been done

3. In your Methods section, please provide additional location information of the collection sites, including geographic coordinates for the data set if available.

The geographic coordinates have been added.

4. Thank you for stating the following in the Acknowledgments Section of your manuscript:

"The performance of this study and the writing of the manuscript was financed by Equinor AS. We are particularly grateful for their role in the study design, discussions of the results, decision to publish, discussions and comments on the manuscript made by Tone K. Frost and Ingunn Nilsen from Equinor AS. NORCE Norwegian Research Centre AS contributed also with internal financial support to the final writing of the manuscript."

We note that you have provided funding information that is not currently declared in your Funding Statement. However, funding information should not appear in the Acknowledgments section or other areas of your manuscript. We will only publish funding information present in the Funding Statement section of the online submission form. 
Please remove any funding-related text from the manuscript and let us know how you would like to update your Funding Statement. Currently, your Funding Statement reads as follows:

"The performance of this study and the writing of the manuscript was financed by Equinor AS. We are particularly grateful for their role in the study design, discussions of the results, decision to publish, discussions and comments on the manuscript made by Tone K. Frost and Ingunn Nilsen from Equinor AS. NORCE Norwegian Research Centre AS contributed also with internal financial support to the final writing of the manuscript."

Please include your amended statements within your cover letter; we will change the online submission form on your behalf.

The Acknowledgments section of the manuscript has been slightly modified. The Funding Statement has been revised too.

5. Thank you for stating the following in the Financial Disclosure section:

"This study and the writing of the manuscript was financed by Equinor AS under contract #4590073409. We are particularly grateful for their role in the study design, discussions of the results, decision to publish, discussions and comments on the manuscript made by Tone K. Frost and Ingunn Nilsen from Equinor AS."

This has been added.

We note that you received funding from a commercial source: Equinor AS
Please provide an amended Competing Interests Statement that explicitly states this commercial funder, along with any other relevant declarations relating to employment, consultancy, patents, products in development, marketed products, etc.

Within this Competing Interests Statement, please confirm that this does not alter your adherence to all PLOS ONE policies on sharing data and materials by including the following statement: "This does not alter our adherence to PLOS ONE policies on sharing data and materials.” (as detailed online in our guide for authors <http://journals.plos.org/plosone/s/competing-interests>).  If there are restrictions on sharing of data and/or materials, please state these. Please note that we cannot proceed with consideration of your article until this information has been declared.

There is no competing interests or conflicts with the funder (Equinor AS) regarding any element of the experimental work of this research, nor used of any products or patents that require a specific statement. None of the authors have relevant declarations relating to employment or relation with the funder. We confirm that data and material from this research can be shared. "This does not alter our adherence to PLOS ONE policies on sharing data and materials.”

6. In your Data Availability statement, you have not specified where the minimal data set underlying the results described in your manuscript can be found. PLOS defines a study's minimal data set as the underlying data used to reach the conclusions drawn in the manuscript and any additional data required to replicate the reported study findings in their entirety. All PLOS journals require that the minimal data set be made fully available. For more information about our data policy, please see <http://journals.plos.org/plosone/s/data-availability>.

Upon re-submitting your revised manuscript, please upload your study’s minimal underlying data set as either Supporting Information files or to a stable, public repository and include the relevant URLs, DOIs, or accession numbers within your revised cover letter. For a list of acceptable repositories, please see <http://journals.plos.org/plosone/s/data-availability#loc-recommended-repositories>. Any potentially identifying patient information must be fully anonymized.

The data used in the paper are now uploaded in Dryad datadryad.org/stash/dashboard with DOI <https://doi.org/10.5061/dryad.t76hdr827> The dataset will now remain private until the manuscript has been accepted when the curation process can start .

7. PLOS requires an ORCID iD for the corresponding author in Editorial Manager on papers submitted after December 6th, 2016. Please ensure that you have an ORCID iD and that it is validated in Editorial Manager. To do this, go to ‘Update my Information’ (in the upper left-hand corner of the main menu), and click on the Fetch/Validate link next to the ORCID field. This will take you to the ORCID site and allow you to create a new iD or authenticate a pre-existing iD in Editorial Manager.

This is now done and the ORCID-iD number is <https://orcid.org/0000-0002-2760-2998>

8. We note that Figure 6 in your submission contain copyrighted images. All PLOS content is published under the Creative Commons Attribution License (CC BY 4.0), which means that the manuscript, images, and Supporting Information files will be freely available online, and any third party is permitted to access, download, copy, distribute, and use these materials in any way, even commercially, with proper attribution. For more information, see our copyright guidelines: <http://journals.plos.org/plosone/s/licenses-and-copyright>.

Figure 6 is an original picture taken by us and not associated to any copyrighted image. Hence, we do not see the need to any particular copyright restriction related to this figure.

9. Please include captions for your Supporting Information files at the end of your manuscript, and update any in-text citations to match accordingly. Please see our Supporting Information guidelines for more information: <http://journals.plos.org/plosone/s/supporting-information>.

This is included. See revised manuscript

**Reviewer's Responses to Questions**

Reviewer #1: I reviewed the ms entitled “Identification of tolerance levels on the cold-water coral Desmophyllum pertusum (Lophelia pertusa) from realistic exposure conditions to suspended bentonite, barite and drill cutting particles” by Thierry Baussant et al. This study explored the tolerances of a CWC (Desmophyllum pertusum syn. Lophelia pertusa) to suspended particle concentration from the oil industry, particularly drill cutting particles. I am aware that the information about this topic is still scarce, thus, this is an important contribution to the general topic of CWC’s and its vulnerability to offshore oil-drilling operations. Overall, the manuscript is well written, technically sound, and the data support the conclusions. Furthermore, I can see interesting findings, particularly in the tolerance of this species to those additional stressors. I am concerned with some of their approaches in the data analysis and there is some missing information in the way how the replicates were treated (replicates for experimental design, decisions made for pooling data, etc). However, I consider these additions to be minor and easily incorporated into the ms.

## Introduction

The introduction is well written and provide with the enough information for the study. It has a clear and stated hypothesis.

Good. Thank you !

Line 91: Should be mg l-1?

Corrected

Methods

Line 183-189: Please provide more information about the measurement equipment: salinity and temperature with precision and accuracy of the measurement.

This information has now been added in the text of the revised version

*CTD model AquaTroll 100, In-Situ Inc, USA*

*Salinity accuracy: ±0.5-1% of reading, + 1 μS/cm when reading < 80,000 μS/cm and resolution: 0.1 μS/cm;*

*Temperature accuracy ± 0.1°C and resolution ~ 0.01°C*

Line 147 – line 195: Wrong citation format

This has been corrected

Line 206 – 210: Is this a standardize methodology? If so, please provide with a cite for this method.

No, not exactly. As far as I know, there is no standardize methodology for measuring particle concentration in the water but it is common to use weighing on pre-weighted GF/F filters after filtration or turbidity/nephelometry measurements with the use of a standard curve established with a series of preset concentrations. The method is for example used in Larsson et al. (2013) and Baussant et al. (2018). These references are added in the text now.

Coral experiments and measurements

Line 227-230: 1) It is not clear to me how you got dry-weight from B-W, since B-W means to weight the fragments in the water.2) Also, I understand DW was used for standardization of % growth rate, but also to quantify growth rates? I am a little confused about this. Please clarify.

1) Please see paper “Spencer Davies, P. Short-term growth measurements of corals using an accurate buoyant weighing technique. *Marine Biology* **101,** 389–395 (1989). <https://doi.org/10.1007/BF00428135>” for a full explanation of the calculation of skeleton DW based on skeleton Buoyancy weight. The skeletal weight of the coral is estimated from the buoyant weight in seawater. The mean density of the skeleton has been determined in previous coral work.
2) Yes, this is correct. DW was used for calculation (not standardization)of growth rate from which % growth rate was obtained. Once skeleton DW of all coral nubbins was calculated, the values were used to normalize respiration rate calculated as mgO2 respired/h/gDW skeleton at each time point and in the calculation of % growth rate (%skeleton growth/day during the experimental period from T0 to T1 and T1 to T2) as well.

The text has been slightly modified to reflect these methodological aspects

Line 230: I know surface area is better than DW for standardization of respiration rates. Please provide a reasoning why using DW instead of SA? Can you cite previous work for this?

As far as we know, both ways of standardization of respiration rates are used and accepted. See for example Reynaud and Ferrier-Pages 2019; Dodds et al. 2007; Larsson et al. 2013; Baussant et al. 2017. As the authors of the present paper have normalized respiration rates to DW in previous work, we chose to use the same normalization method in the present article too.

These references have been added to the corresponding line.

Data analysis

lines 273 – 277: “replicate tank nested factor was tested using a Kruskal-Wallis analysis (p<0.05), and the data from the replicate tanks were pooled if the assumption of equal distribution was verified”. Please be more specific about this analysis. Did you pool all your fragments from the different cones? or Cones were treated as independent?

Yes, the fragments of corals from the different cones were pooled after the assumption of equal data distribution in the different cones was verified using the Kruskal-Wallis analysis. The H0 hypothesis tested was that the distribution of all variables measured in corals was the same across the different cones. Below an example of output from the KW analysis test (SPSS statistical software) for respiration data measured in the DC exposure.

The text has been slightly modified to make that clearer.

Line 286 – 290. The authors state that for growth rate and polyp mortality measurements, “the
ANOVA assumptions were violated, and the effects of DC and BABE treatments were analyzed using a Kruskal–Wallis test on ranks at each sampling time, i.e. T1, T2 (p<0.05)”. 1)Did you apply corrections for multiple comparison? i.e. Bonferroni correction? Also, it is not clear to me what do you mean with “Kruskal–Wallis test on ranks at each sampling time, i.e. T1, T2 (p < 0.05)? T1 and T2 were significantly different? are those values part of the results? 2) The same as for line 290: Were values pooled or not pooled?

We have modified the text to clarify better this point. Here is how the text reads now:

1) Prior to analysis, the nested factor “replicate tank” was tested for equal distribution of coral measurement variance across the replicate tanks and, given the few number of coral nubbins in each tank (n=4 to 6), a non-parametric analysis was used. We found no significant difference in coral measurement variance across the different cones (Kruskal-Wallis rank test, p<0.05) and coral data from the different replicate tanks (n=3) of treatment and control were pooled. Then a Kolmogorov-Smirnov was applied to test for Gaussian distribution and the assumption of equal variance (homoscedasticity) for the different treatment groups was verified (Levene’s test) for p>0.05. These assumptions were met for all measurements but for coral growth rate and polyp mortality for which a Kruskal–Wallis rank non-parametric test was applied, tested for each sampling time i.e. for T1 and T2 (p < 0.05) and a Mann-Whitney 2-sample test used as post-hoc analysis. For all other measurements, a repeated ANOVA was applied to test mean differences with treatment concentration as a between-subjects factor and sampling time as a within-subject factor. When the assumption of sphericity was not met (Mauchly’s test, p < 0.05), p-values were adjusted for repeated time measurements using Greenhouse–Geisser epsilon and Hunyh–Feldt epsilon. A post hoc analysis with Tukey’s honest significant difference (Tukey’s HSD) test was applied to identify differences between means in treatment and control groups. The output of the Kruskal-Wallis test is part of the results and now added in the text.
2) The polyp mortality data were also tested for equal distribution between replicate cones and were pooled as commented above in the data analysis section. As polyp mortality was measured during days of feeding and days without feeding, we also pooled polyp mortality from days with and without feeding to increase the number of observations when there was no significant differences or used only days with feeding if there was a significant difference. This is now modified in the text as “Polyp mortality scoring determined during the days with and without feeding were pooled if no significant difference was observed or otherwise only days with feeding were used if there was a significant difference (Wilcoxon signed rank test, p≤0.5).”

Line 264 – check for typo

Outliers and extreme values were not removed from the overall data analysis, taking into
Corrected in the text

Line 288 – check for typo
the ANOVA assumptions were violated, and the effects of DC and BABE treatments were analyzed using a Kruskal–Wallis rank test for each sampling time separately, i.e. for T1 and T2 (p < 0.05). Corrected in the text

Results and Discussion

Respiration rates: “Under stress conditions, such as with the presence of solid particles in the water, one could expect an increase in respiration rate consequent to the higher metabolic activity resulting from the removal of particles by polyps and mucus secretion”. Could you provide a better explanation on why respiration rates are unaffected under such stressful conditions? Why is this lack of sensitivity?

It is difficult to say but lack of change in respiration rate after particle exposure on deep-sea corals has been reported before as well, even for longer exposure time (see for example Larsson et al., 2013; Baussant et al., 2018). Here the corals were exposed to even shorter exposure (5 days). It has been suggested that the lack of change in respiration rate might be due to optimal coral feeding in laboratory experiments, masking the real effect of stressors on the respiration rates (Reynaud and Ferrier-Pagès, 2019). I do not believe this is a lack senstivity in that measurement since we do measure change when the corals are deprived from food for a long period for example (Larsson et al., 2013; Baussant et al., 2017).

The text has been slightly adapted in the corresponding section

Polyp mortality:

Line 506 – 510: “In the DC experiment, we found that polyp mortality determination measured during days with addition of food particles and days without food addition was equal (Wilcoxon signed rank test, p=0.103). Hence, data from days of feeding and no feeding were pooled to test differences in polyp mortality resulting from DC treatments at T1 and T2”. As stated above, I am a little bit concern about the procedure of pooling data. Do you have any other previous work that validate your assumption? Normally, a conservative estimate of the p-value for pooling data should be above p>0.25, however, I might be outdated about this procedure. Please provide a reasoning.

I am sorry, we are not aware that a conservative estimate of the p-value for pooling data should be above p>0.25. We have used this pooling approach in two former papers (Baussant et al., 2017 and 2018) with corals in a similar setup and used the same methodology and p value to justify pooling.

Line 531 – 533: I would start the paragraph with the actual value of % polyp mortality found in your study, and then compared to the other studies. I know you referenced the figure 7, but it would be helpful for the reader to find the actual values found in your study written in the paragraph.

Thank you for this comment. This makes sense. The actual value of % polyp mortality found in your study has been moved at the front of the paragraph and the comparison to other studies at the end of the paragraph of this section.

Line 551 – 554: It looks speculative. Does not support/explain your results.

“However, the coenosarc condition was not checked accurately for these branches at the start of the experiment, so it is difficult to conclude if coenosarc condition at start of the experiment or deterioration during the exposure is explaining the relatively high polyp mortality observed in the DC experiment.”

This sentence was removed and the text of this section was slightly adjusted.

Figure 2. What is the n for each treatment? See also comments in the methods section.

The n was added on the legend of each figure to reflect that the data from each replicate tank for treatment and control group were pooled at each sampling time.
